# Rural–Urban Disparities in State-Level Diabetes Prevalence Among US Adults, 2021

**DOI:** 10.5888/pcd22.240199

**Published:** 2025-01-16

**Authors:** Olga Khavjou, Zohra Tayebali, Pyone Cho, Kristopher Myers, Ping Zhang

**Affiliations:** 1 RTI International, Research Triangle Park, North Carolina; 2 Centers for Disease Control and Prevention, Atlanta, Georgia; 3 Goldbelt C6, LLC, Chesapeake, Virginia

## Abstract

**Introduction:**

We assessed state-level disparities in diabetes prevalence among adults in rural and urban areas in the United States.

**Methods:**

We estimated state-specific diabetes prevalence in rural and urban areas in 41 states with applicable data from the 2021 Behavioral Risk Factor Surveillance System. Rural areas were defined based on the 2013 National Center for Health Statistics Urban–Rural Classification Scheme. We estimated diabetes odds ratios (ORs) in rural versus urban areas in each state by using logistic regressions adjusted for sociodemographic characteristics and obesity status. Analyses were conducted in 2023.

**Results:**

In rural areas, diabetes prevalence was 14.3%, ranging from 8.4% in Colorado to 21.3% in North Carolina. In urban areas, the prevalence was 11.2%, ranging from 6.9% in Colorado to 15.5% in West Virginia. Unadjusted diabetes ORs in rural versus urban areas were significant (*P* < .05) and greater than 1 for 19 states. After adjusting for age, sex, race, and ethnicity, the ORs were significant and greater than 1 for 7 states (Florida, Illinois, Kentucky, Maryland, North Carolina, Oregon, and Virginia). With additional adjustment for education, income, and obesity status, diabetes ORs in rural versus urban areas remained significant and greater than 1 for 2 states (North Carolina and Oregon).

**Conclusion:**

Our findings reveal significant geographic disparities in diabetes prevalence between rural and urban areas in 19 states. The differences in most states may have been explained by rural–urban differences in sociodemographic characteristics and obesity rates. Our findings could inform decision makers to identify effective ways to reduce rural–urban disparities within states.

**SUMMARY**

**What is already known on this topic?**

Prevalence of diabetes is 9% to 17% higher in rural areas than in urban areas. Common risk factors of diabetes, such as age, race, ethnicity, income, and obesity may explain the rural–urban disparities.

**What is added by this report?**

This study examines rural–urban disparities in diabetes prevalence across states, providing a better understanding of the geographic distribution and underlying attributes associated with higher diabetes prevalence among people who live in rural areas.

**What are the implications for public health practice?**

Identifying drivers of rural–urban disparities in diabetes prevalence by state underscores the need for planned interventions and resources to address diabetes in rural communities.

## Introduction

Diabetes is a serious chronic health condition and is a major contributor to heart disease, kidney failure, stroke, vascular disorders, and vision loss (1). In 2021, the Centers for Disease Control and Prevention (CDC) reported that more than 38 million adults were living with diabetes (2). Diabetes has been identified as one of the top 10 Healthy People 2030 priorities for the rural United States (3,4). Public health practitioners, researchers, and policymakers deemed diabetes an important health priority to address in the coming decade to close the rural–urban divide (4).

Prevalence of diabetes has been reported from 9% to 17% higher in rural areas than in urban areas (5,6). Demographic characteristics, socioeconomic status, neighborhood characteristics, physical environment, food environment, prevalence of health behavior risk factors, and chronic disease prevention efforts are potential factors that explain rural–urban differences in prevalence of diabetes (7,8). Specifically, O’Connor and Wellenius examined rural–urban disparities in diabetes prevalence at the national level and found that age, sex, race, ethnicity, income, and obesity were factors that contributed to the differences (6). However, to our knowledge, the rural–urban disparities in diabetes prevalence by state have not been examined systematically.

In 2016, CDC released the Diabetes State Burden Toolkit, reporting data on the health, economic, and mortality burden of diabetes in each state and the District of Columbia (DC). In 2024, the toolkit was updated with more recently available data and expanded to report diabetes outcomes by urbanicity status (https://nccd.cdc.gov/Toolkit/DiabetesBurden). The goal of the update to the toolkit was to meet information needs of state health officials and other organizations. The objectives of our study were to 1) assess the magnitude of rural–urban differences in diabetes prevalence by state as reported in the toolkit, and 2) identify the underlying factors that may be contributing to the rural–urban disparities at the state level.

## Methods

### Source of data

We used data from the 2021 Behavioral Risk Factor Surveillance System (BRFSS) to estimate diabetes prevalence in each state. BRFSS is a yearly, state-based, cross-sectional telephone interview survey sponsored by CDC and conducted by state health departments. It covers the civilian noninstitutionalized adult population aged 18 years or older in each of the 50 states and the District of Columbia (DC). BRFSS collects prevalence data regarding health-related risk behaviors, chronic health conditions, and preventive health care practices among US adults. Response rates for the BRFSS vary by state. The median survey response rate in the 2021 BRFSS for states included in this analysis was 46.4% and ranged from 23.5% to 60.5% (9).

We downloaded the 2021 BRFSS data file that included all states, except Florida, directly from the BRFSS website. The 2021 Florida BRFSS data set was requested and obtained from the Florida Department of Health.

### Study population

We identified people with diabetes as those who answered yes to the survey question, “Has a doctor, nurse, or other health professional ever told you that you had diabetes?” The estimates reported in this analysis are for both type 1 and type 2 diabetes combined because of data limitations. We excluded survey responses with missing diabetes status (n = 989). We applied the BRFSS sample weights and calculated the weighted percentage of adults with self-reported diagnosed diabetes in each state.

In BRFSS, rural or urban status of the county where the respondent resides is defined by using the 2013 National Center for Health Statistics (NCHS) Urban–Rural Classification Scheme for US counties. The scheme states that urban counties include large central metropolitan, large fringe metropolitan, medium metropolitan, small metropolitan, and micropolitan counties (10). Rural counties include noncore counties (ie, nonmetropolitan counties that do not qualify as micropolitan). In BRFSS, the rural or urban status is assigned based on the county Federal Information Processing Standards codes rather than respondent self-reported information on whether they reside in a rural or urban county.

Seven states (Connecticut, Delaware, Hawaii, Massachusetts, New Hampshire, New Jersey, and Rhode Island) and DC did not have any respondents from rural counties in the 2021 BRFSS. In 2 other states (California and Nevada), 2021 BRFSS data for diabetes prevalence in rural counties did not meet the NCHS data presentation standard of the minimum relative confidence interval width (11,12). Thus, we excluded these 9 states and DC from this analysis (n = 60,233). The final BRFSS analysis sample included 378,504 observations.

### Analysis methods

We calculated prevalence of diabetes in 41 states where data were available for both rural and urban areas. To compare diabetes prevalence between rural and urban areas, we calculated the odds ratios (ORs) of diabetes in rural versus urban areas to help understand the likelihood of diabetes occurring in one area compared with the other. Specifically, we ran separate logistic regressions for each state and for 41 states combined to estimate the ORs of having diabetes for people residing in rural versus urban counties. An OR greater than 1 indicates a higher likelihood of diabetes in rural areas than in urban areas. An OR less than 1 indicates a lower likelihood of diabetes in rural areas than in urban areas.

We ran a series of models controlling for different factors. The first set of regressions produced unadjusted ORs, including only the rural or urban status indicator and no controls for any other characteristics. Then, we estimated 3 other sets of regression models and produced adjusted ORs, one controlling for age and sex, the second controlling for age, sex, race, and ethnicity, and the third controlling for age, sex, race, ethnicity, income, education, and obesity status. These adjusted regression results allow us to assess whether the differences in likelihood of diabetes between rural and urban areas can be explained by the differences in the sociodemographic composition and obesity rates of the populations living in rural and urban areas. All regression models were estimated by applying BRFSS sample weights to account for the complex survey design.

We controlled for age in years as a continuous variable. Race and ethnicity categories included 4 categories: non-Hispanic White, non-Hispanic Black, Hispanic, and non-Hispanic other races (which included Asian, American Indian and Alaska Native, and multiracial). We categorized income into 3 groups based on the annual household income: low income (<$35,000), middle income ($35,000 to $74,999), and high income (≥$75,000). These income categories were obtained by using Healthy People 2020 groupings and categorizations but collapsing BRFSS’s 2 lowest income groups into 1 group (low income) and the middle and near-high income groups into another group (middle income) to ensure sufficient sample sizes (13). We defined educational attainment based on the highest grade or years of school completed: less than high school graduate, high school graduate, and more than high school graduate. Lastly, obesity status was determined by body mass index (BMI), calculated as weight in kilograms divided by the square of height in meters. The categories were underweight or normal weight (BMI <25), overweight (BMI 25 to 29.9), and obese (BMI ≥30).

We considered results significant in a specific state when the probability of a difference in likelihood of diabetes between rural and urban areas occurring by chance was less than 5% in that state. We conducted our analyses in 2023 by using Stata version 17 (StataCorp LLC).

## Results

### Unadjusted results

Across the 41 states included in this analysis, diabetes prevalence in 2021 was 14.3% (95% CI, 13.5%–15.0%) in rural areas and 11.2% (95% CI, 10.9%–11.4%) in urban areas (Table 1). Adults living in rural areas were, on average, older, had lower household incomes and lower levels of education, were more likely to be non-Hispanic White, and were less likely to be non-Hispanic Black, Hispanic, and non-Hispanic other races than adults living in urban areas.

**Table 1:** Demographic Characteristics by Rural and Urban Areas, 41 US States^a^, Behavioral Risk Factor Surveillance System, 2021

Characteristic	Rural Areas, Mean (95% CI)	Urban Areas, Mean (95% CI)
**Self-reported diagnosis of diabetes, %**	**14.3 (13.5–15.0)**	**11.2 (10.9–11.4)**
**Age, y**	**51.5 (51.1–51.9)**	**47.9 (47.7–48.0)**
**Annual household income, $, %**		
Low (<35,000)	30.2 (29.3–31.2)	23.1 (22.7–23.4)
Middle (35,000 to 74,999)	26.2 (25.4–27.0)	23.2 (22.9–23.6)
High (≥75,000)	43.6 (42.6–44.5)	53.7 (53.3–54.1)
**Sex, %**		
Male	49.9 (49.0–50.9)	48.6 (48.2–49.0)
Female	50.1 (49.1–51.0)	51.4 (51.0–51.8)
**Body weight^b^ category, %**		
Underweight or normal weight (<25)	24.6 (23.7–25.4)	28.3 (27.9–28.6)
Overweight (25 to 29.9)	30.5 (29.6–31.4)	30.1 (29.8–30.5)
Obese (≥30)	35.0 (34.0–36.0)	29.6 (29.2–29.9)
**Race and ethnicity, %**		
Non-Hispanic White	80.7 (79.8–81.7)	64.7 (64.3–65.1)
Non-Hispanic Black	7.9 (7.3–8.4)	13.5 (13.2–13.8)
Hispanic	6.7 (5.8–7.6)	14.8 (14.4–15.2)
Non-Hispanic other races	4.7 (4.4–5.0)	7.0 (6.8–7.2)
**Education level, %**		
Less than high school graduate	14.8 (13.9–15.6)	11.1 (10.8–11.5)
High school graduate	37.0 (36.0–38.0)	27.6 (27.3–28.0)
More than high school graduate	47.8 (46.8–48.7)	60.6 (60.2–61.0)

a The states of California, Connecticut, Delaware, Hawaii, Massachusetts, Nevada, New Hampshire, New Jersey, and Rhode Island and the District of Columbia were excluded from this analysis because of insufficient or unreliable data.

b Body weight category was determined by calculating weight in kilograms divided by the square of height in meters.

Prevalence of diabetes in rural areas varied widely across states, ranging from 8.4% (95% CI, 6.1%–10.7%) in Colorado to 21.3% (95% CI, 15.9%–26.7%) in North Carolina, with the all-state median of 13.2% (Table 2). A total of 11 states in the Southeast (Alabama, Florida, Georgia, Kentucky, Louisiana, Mississippi, North Carolina, South Carolina, Tennessee, Virginia, West Virginia), plus Illinois, Oregon, and Pennsylvania, had the highest diabetes prevalence rates in rural areas. These 14 states had prevalences of 15.8% or higher and were in the top third of the distribution (ie, upper tertile). Six states in the Midwest (Iowa, Michigan, Minnesota, Nebraska, Ohio, and Wisconsin), 6 states in the West (Alaska, Colorado, Idaho, Montana, Utah, and Wyoming), along with Maine and Vermont, had the lowest diabetes prevalence rates in rural areas. These 14 states had prevalences of 11.8% or higher and were in the bottom third of the distribution (ie, lower tertile).

**Table 2:** Prevalence of Diagnosed Diabetes in Rural and Urban Counties by State^a^, Behavioral Risk Factor Surveillance System, 2021

State	Rural areas		Urban areas	
	N^b^	% (95% CI)	N^b^	% (95% CI)
Alabama	625	15.9 (12.5–19.4)	3,955	14.9 (13.5–16.3)
Alaska	2,149	9.8 (7.9–11.6)	3,330	7.8 (6.6–9.1)
Arizona	573	12.7 (7.8–17.7)	10,060	11.0 (10.2–11.8)
Arkansas	1,227	15.7 (13.1–18.2)	4,134	11.7 (10.5–12.9)
Colorado	723	8.4 (6.1–10.7)	9,738	6.9 (6.3–7.5)
Florida	1,376	17.2 (13.0–21.4)	6,539	10.8 (9.4–12.3)
Georgia	981	17.3 (14.1–20.6)	7,186	11.9 (10.8–12.9)
Idaho	805	11.5 (8.9–14.1)	5,964	9.6 (8.7–10.4)
Illinois	194	18.1 (10.9–25.3)	3,004	10.4 (9.1–11.8)
Indiana	617	14.1 (10.9–17.3)	9,285	12.0 (11.3–12.7)
Iowa	2,721	11.2 (9.8–12.6)	6,890	9.2 (8.4–10.0)
Kansas	3,084	12.0 (10.7–13.3)	14,450	10.9 (10.3–11.5)
Kentucky	1,615	16.7 (14.1–19.4)	3,802	13.0 (11.6–14.4)
Louisiana	329	15.8 (9.4–22.3)	4,760	13.5 (12.2–14.7)
Maine	6,139	11.5 (10.4–12.5)	5,643	10.0 (9.0–10.9)
Maryland	519	13.7 (10.1–17.3)	15,071	11.0 (10.4–11.7)
Michigan	666	10.4 (7.9–12.8)	8,731	10.8 (10.0–11.6)
Minnesota	1,892	10.7 (9.0–12.3)	14,040	8.8 (8.2–9.3)
Mississippi	1,276	16.4 (13.9–19.0)	3,140	14.9 (13.3–16.4)
Missouri	3,322	13.2 (11.7–14.6)	8,923	11.0 (10.1–11.9)
Montana	2,958	10.7 (9.3–12.1)	3,277	7.9 (6.9–8.8)
Nebraska	4,976	10.7 (9.7–11.6)	9,923	9.4 (8.7–10.1)
New Mexico	335	15.4 (11.0–19.8)	6,022	13.1 (11.9–14.2)
New York	4,225	12.1 (10.1–14.1)	34,753	11.4 (10.8–12.0)
North Carolina	377	21.3 (15.9–26.7)	4,555	12.1 (10.9–13.3)
North Dakota	2,406	12.7 (11.0–14.5)	3,493	8.4 (7.4–9.5)
Ohio	1,136	11.8 (9.5–14.1)	13,140	12.6 (11.8–13.4)
Oklahoma	1,012	14.3 (11.8–16.8)	4,428	12.6 (11.4–13.8)
Oregon	150	20.4 (11.2–29.6)	5,214	9.2 (8.3–10.2)
Pennsylvania	238	17.1 (9.9–24.3)	6,164	10.9 (9.9–11.9)
South Carolina	908	17.8 (14.1–21.5)	9,122	13.5 (12.6–14.4)
South Dakota	2,295	12.6 (8.2–16.9)	4,972	10.3 (8.6–12.0)
Tennessee	661	18.5 (14.5–22.5)	4,110	13.4 (12.1–14.7)
Texas	403	13.2 (7.2–19.3)	10,383	11.4 (10.4–12.4)
Utah	1,227	8.8 (6.8–10.8)	9,373	7.9 (7.3–8.5)
Vermont	2,311	9.0 (7.5–10.5)	4,256	8.3 (7.2–9.5)
Virginia	1,347	16.4 (13.5–19.2)	8,511	10.9 (10.0–11.7)
Washington	985	12.7 (8.5–16.9)	12,141	8.6 (8.0–9.2)
West Virginia	1,453	17.0 (14.7–19.3)	5,281	15.5 (14.4–16.7)
Wisconsin	1,635	10.6 (8.3–12.9)	4,463	8.9 (7.8–10.0)
Wyoming	1,399	9.1 (7.3–10.9)	3,008	8.7 (7.4–9.9)
Total	63,270	14.3 (13.5–15.0)	315,234	11.2 (10.9–11.4)
Median		13.2		10.9

a The states of California, Connecticut, Delaware, Hawaii, Massachusetts, Nevada, New Hampshire, New Jersey, and Rhode Island and the District of Columbia were excluded from this analysis because of insufficient or unreliable data.

b Represents the unweighted number of observations from the 2021 Behavioral Risk Factor Surveillance System.

In urban areas of the 41 states included in the analysis, the diabetes prevalence ranged from 6.9% (95% CI, 6.3%–7.5%) in Colorado to 15.5% (95% CI, 14.4%–16.7%) in West Virginia, with the median of 10.9%.

Unadjusted ORs of diabetes in rural versus urban areas were significant and greater than 1 in the 41 states combined and in 19 individual states (Arkansas, Florida, Georgia, Illinois, Iowa, Kentucky, Maine, Minnesota, Missouri, Montana, Nebraska, North Carolina, North Dakota, Oregon, Pennsylvania, South Carolina, Tennessee, Virginia, and Washington) (Table 3). Across these 19 states, the unadjusted ORs ranged from 1.1 (95% CI, 1.0–1.3) in Nebraska to 2.5 (95% CI, 1.4–4.5) in Oregon.

**Table 3:** Odds Ratios of Diabetes in Rural Versus Urban Areas by State^a^, Behavioral Risk Factor Surveillance System, 2021

State	Model 1: unadjusted		Model 2: adjusted for age and sex		Model 3: adjusted for age, sex, race, ethnicity		Model 4: adjusted for age, sex, race, ethnicity, income, education, obesity status	
	OR (95% CI)	*P* value	OR (95% CI)	*P* value	OR (95% CI)	*P* value	OR (95% CI)	*P* value
Alabama	1.1 (0.8–1.4)	.59	1.0 (0.7–1.3)	.75	0.9 (0.7–1.3)	.70	0.9 (0.6–1.1)	.29
Alaska	1.3 (1.0–1.7)	.08	1.2 (0.9–1.5)	.29	1.1 (0.8–1.5)	.38	1.1 (0.8–1.5)	.52
Arizona	1.2 (0.7–1.8)	.48	1.1 (0.7–1.8)	.63	0.8 (0.5–1.3)	.48	0.7 (0.4–1.1)	.14
Arkansas	1.4^b^ (1.1–1.8)	.00	1.2 (1.0–1.5)	.12	1.2 (1.0–1.6)	.09	1.2 (0.9–1.5)	.13
Colorado	1.2 (0.9–1.7)	.20	1.0 (0.7–1.4)	.99	1.0 (0.7–1.4)	.96	1.0 (0.7–1.4)	.86
Florida	1.7^b^ (1.2–2.4)	.00	1.4 (1.0–2.0)	.07	1.5^b^ (1.0–2.1)	.03	1.2 (0.9–1.8)	.26
Georgia	1.6^b^ (1.2–2.0)	.00	1.2 (0.9–1.5)	.15	1.3 (1.0–1.6)	.08	1.1 (0.8–1.5)	.51
Idaho	1.2 (0.9–1.6)	.14	1.0 (0.8–1.3)	.93	1.0 (0.8–1.3)	.92	0.9 (0.7–1.2)	.45
Illinois	1.9^b^ (1.1–3.1)	.01	1.6 (0.9–2.7)	.12	1.8^b^ (1.0–3.2)	.03	1.7 (1.0–3.0)	.06
Indiana	1.2 (0.9–1.6)	.18	1.1 (0.8–1.4)	.59	1.2 (0.9–1.6)	.30	1.1 (0.8–1.5)	.50
Iowa	1.2^b^ (1.0–1.5)	.01	1.0 (0.9–1.2)	.66	1.1 (0.9–1.3)	.33	1.0 (0.8–1.2)	.95
Kansas	1.1 (1.0–1.3)	.12	0.9 (0.8–1.1)	.37	1.0 (0.8–1.1)	.68	0.9 (0.7–1.0)	.05
Kentucky	1.3^b^ (1.1–1.7)	.01	1.2 (1.0–1.5)	.11	1.3^b^ (1.0–1.6)	.04	1.1 (0.9–1.4)	.46
Louisiana	1.2 (0.7–2.0)	.45	1.0 (0.6–1.8)	.97	1.0 (0.5–1.9)	.97	0.9 (0.5–1.7)	.82
Maine	1.2^b^ (1.0–1.4)	.04	1.0 (0.9–1.2)	.61	1.0 (0.9–1.2)	.60	0.9 (0.8–1.1)	.46
Maryland	1.3 (0.9–1.7)	.12	1.2 (0.9–1.6)	.16	1.4^b^ (1.1–1.9)	.02	1.2 (0.9–1.6)	.25
Michigan	1.0 (0.7–1.3)	.74	0.8 (0.6–1.1)	.12	0.9 (0.7–1.2)	.50	0.8 (0.6–1.1)	.22
Minnesota	1.2^b^ (1.0–1.5)	.02	1.1 (0.9–1.3)	.58	1.1 (0.9–1.3)	.31	1.0 (0.8–1.2)	.81
Mississippi	1.1 (0.9–1.4)	.28	1.0 (0.8–1.3)	.72	1.1 (0.8–1.3)	.63	1.0 (0.8–1.3)	.91
Missouri	1.2^b^ (1.1–1.4)	.01	1.1 (0.9–1.3)	.44	1.1 (1.0–1.4)	.11	1.0 (0.8–1.2)	.90
Montana	1.4^b^ (1.2–1.7)	.00	1.2 (1.0–1.5)	.07	1.1 (0.9–1.4)	.29	1.1 (0.9–1.3)	.55
Nebraska	1.1^b^ (1.0–1.3)	.04	0.9 (0.8–1.1)	.26	1.0 (0.9–1.1)	1.00	0.9 (0.8–1.1)	.30
New Mexico	1.2 (0.9–1.7)	.28	1.0 (0.7–1.4)	.85	1.0 (0.7–1.5)	.84	1.0 (0.7–1.4)	.95
New York	1.1 (0.9–1.3)	.52	0.9 (0.8–1.2)	.58	1.2 (1.0–1.5)	.09	1.0 (0.8–1.3)	.71
North Carolina	2.0^b^ (1.4–2.8)	.00	1.6^b^ (1.1–2.3)	.01	1.7^b^ (1.1–2.4)	.01	1.5^b^ (1.0–2.1)	.04
North Dakota	1.6^b^ (1.3–1.9)	.00	1.3^b^ (1.0–1.6)	.04	1.1 (0.9–1.4)	.22	1.1 (0.9–1.4)	.32
Ohio	0.9 (0.7–1.2)	.51	0.8 (0.7–1.1)	.12	0.9 (0.7–1.1)	.21	0.8^b^ (0.6–1.0)	.04
Oklahoma	1.2 (0.9–1.5)	.21	1.0 (0.8–1.2)	.75	1.0 (0.8–1.2)	.84	0.9 (0.7–1.2)	.68
Oregon	2.5^b^ (1.4–4.5)	.00	2.2^b^ (1.2–4.1)	.01	2.0^b^ (1.1–3.7)	.02	2.5^b^ (1.3–4.8)	.00
Pennsylvania	1.7^b^ (1.0–2.8)	.04	1.4 (0.8–2.3)	.24	1.5 (0.9–2.6)	.11	1.4 (0.8–2.3)	.22
South Carolina	1.4^b^ (1.1–1.8)	.01	1.3 (1.0–1.8)	.05	1.2 (0.9–1.6)	.24	1.1 (0.8–1.5)	.42
South Dakota	1.2 (0.8–1.9)	.32	1.0 (0.6–1.5)	.92	0.8 (0.5–1.3)	.46	0.8 (0.5–1.2)	.25
Tennessee	1.5^b^ (1.1–1.9)	.01	1.2 (0.9–1.6)	.33	1.2 (0.9–1.7)	.17	1.1 (0.8–1.5)	.42
Texas	1.2 (0.7–2.0)	.53	1.1 (0.6–2.0)	.81	1.2 (0.6–2.2)	.61	1.0 (0.5–1.9)	.97
Utah	1.1 (0.9–1.5)	.38	0.9 (0.7–1.2)	.68	1.0 (0.7–1.3)	.90	0.9 (0.7–1.2)	.48
Vermont	1.1 (0.9–1.4)	.48	1.0 (0.8–1.3)	.94	1.0 (0.8–1.3)	.93	1.0 (0.7–1.2)	.71
Virginia	1.6^b^ (1.3–2.0)	.00	1.3^b^ (1.0–1.6)	.04	1.4^b^ (1.1–1.8)	.00	1.2 (0.9–1.5)	.20
Washington	1.5^b^ (1.0–2.3)	.03	1.1 (0.7–1.6)	.80	1.1 (0.7–1.7)	.64	1.1 (0.7–1.6)	.74
West Virginia	1.1 (0.9–1.3)	.25	1.0 (0.8–1.2)	.83	1.0 (0.8–1.2)	.77	0.9 (0.8–1.2)	.60
Wisconsin	1.2 (0.9–1.6)	.17	1.0 (0.8–1.4)	.76	1.2 (0.9–1.6)	.24	1.1 (0.8–1.4)	.58
Wyoming	1.1 (0.8–1.4)	.71	0.9 (0.7–1.1)	.27	0.9 (0.7–1.2)	.35	0.9 (0.7–1.2)	.40
Total	1.3^b^ (1.2–1.4)	.00	1.1^b^ (1.0–1.2)	.00	1.2^b^ (1.1–1.3)	.00	1.1 (1.0–1.1)	.12

Abbreviation: OR, odds ratio.

a The states of California, Connecticut, Delaware, Hawaii, Massachusetts, Nevada, New Hampshire, New Jersey, and Rhode Island and the District of Columbia were excluded from this analysis because of insufficient or unreliable data.

b Indicates significant odds ratios (*P* < .05).

### Adjusted results

After adjusting for age and sex, the ORs of diabetes in rural versus urban areas remained significant and greater than 1 in the 41 states combined and in 4 individual states (North Carolina, North Dakota, Oregon, and Virginia) (Table 3). The ORs across these 4 states ranged from 1.3 (95% CI, 1.0–1.6) in North Dakota to 2.2 (95% CI, 1.2–4.1) in Oregon with a median of 1.5.

After further adjustment for race and ethnicity (in addition to age and sex), the ORs of diabetes in rural versus urban areas were significant and greater than 1 in the 41 states combined and 7 individual states (Florida, Illinois, Kentucky, Maryland, North Carolina, Oregon, and Virginia). The ORs across these 7 states ranged from 1.3 (95% CI, 1.0–1.6) in Kentucky to 2.0 (95% CI, 1.1–3.7) in Oregon with a median of 1.5.

With additional adjustment for income, education, and obesity status, the diabetes OR for the 41 states combined was no longer significant (*P* = .12). However, ORs remained significant and greater than 1 in 2 individual states, namely North Carolina (OR, 1.5; 95% CI, 1.0–2.1) and Oregon (OR, 2.5; 95% CI, 1.3–4.8). In 1 state (Ohio), this additional adjustment resulted in a significant OR of less than 1 (0.77; 95% CI, 0.60–0.98). This finding indicates that once adjusted for age, sex, race, ethnicity, income, education, and obesity status, the likelihood of diabetes was significantly lower in rural areas than in urban areas of Ohio.

## Discussion

We examined the ORs of diabetes in rural versus urban areas at the state level and found that geographic disparities in likelihood of diabetes between rural and urban areas varied across the states. Of the 41 states included in the study, the likelihood of diabetes was significantly higher in rural areas than in urban areas in 19 states. Differences in sociodemographic characteristics and obesity rates may have explained those rural–urban disparities in most states. Our study results could help decision makers at the state level understand the rural–urban differences in diabetes prevalence in their states and identify effective measures to close the rural–urban gaps.

The result that only 4 of 19 states had a significantly higher likelihood of diabetes in rural versus urban areas, after adjusting for age and sex, implies that differences in population composition could be the main driver of the rural–urban differences in diabetes prevalence. For example, older adults are more prone to have diabetes. In 2022, prevalence of diabetes at the national level was 2.4% among adults aged 18 to 44 years and 20.6% among adults aged 75 years or older (14). Adults in rural areas were older than those in urban areas (Table 1) (15,16). Similarly, compared with women, men have higher rates of diabetes and are more likely to live in rural than in urban areas (Table 1) (17,18).

After also adjusting for race and ethnicity, significant differences in likelihood of diabetes between rural and urban areas were observed in 4 additional states. This finding indicates that not considering the racial and ethnic differences between urban and rural areas may mask differences in diabetes prevalence between these populations. This finding is important, especially given the increasing racial and ethnic diversity in rural areas of the US (19). Adults from racial and ethnic minority groups living in rural areas may face additional challenges that their counterparts residing in urban areas do not. The higher prevalence of diabetes among Black people, coupled with limited access to health care services in rural settings, places them at an elevated risk for adverse health outcomes (4,20). Diabetes mortality rates among Black people in rural areas are higher than those among White people living in rural areas, underscoring the need for planned interventions (21,22).

Further adjustment for income, education, and obesity status in our models revealed that likelihood of diabetes remained significantly higher in rural than urban areas in only 2 states. Understanding the factors, specifically sociodemographic characteristics and obesity rates, that contribute to the differences in prevalence of diabetes between rural and urban areas could help develop more tailored interventions for populations in these areas.

Oregon and North Carolina were the 2 states where adjusting for sociodemographic characteristics and obesity status did not fully explain the higher likelihood of diabetes in rural versus urban areas. Further research is needed to understand what other factors, such as rural–urban differences in neighborhood characteristics, food and diet behaviors, physical activity levels, and access to healthy food and prevention efforts, could potentially explain these disparities. Identified barriers for people living in rural communities, especially for getting access to diabetes education and prevention programs, include limited number of providers, longer distance to medical facilities, higher costs, outdated cultural beliefs, lack of transportation, and limited community resources (23,24). More efforts to reduce these barriers may help reduce the overall high burden of diabetes in the rural US.

Results of our analysis aligned with a previous study that used 2008 BRFSS data and demonstrated that, at the national level, rural–urban disparities could be attributed to demographic characteristics and other common risk factors such as income and BMI (6). However, O’Connor and Wellenius found that, at the national level, after adjusting for household income, educational attainment, age, sex, BMI, race, and ethnicity, the likelihood of diabetes was significantly lower in rural areas than in urban areas (OR, 0.94; *P* < .05) (6). Using more recent data, we found that after controlling for these sociodemographic characteristics and obesity status, there were no significant differences in the likelihood of diabetes in rural and urban areas at the national level. At the state level, we found that likelihood of diabetes was significantly lower among respondents living in rural areas than among respondents living in urban areas in 1 state (Ohio). Our findings could indicate the worsening of rural–urban disparities over the last decade.

Our finding that most states with a high prevalence of diabetes were primarily in the Southeast was also consistent with a recent study from 2022 that reported similar geographic trends, indicating that the adults living in the rural South had the highest risk for diabetes (25).

### Strengths and limitations

To our knowledge, this is the first study reporting differences in likelihood of diabetes in rural versus urban areas at the state level. Our findings highlight a higher likelihood of diabetes in rural counties compared with their urban counterparts in most states. This information could help policymakers and public health professionals better understand the diabetes burden in their states.

Our analysis is subject to several limitations. First, the lack of information in BRFSS to distinguish between diabetes types prevented us from generating separate estimates for type 1 and type 2 diabetes. This may hinder identification of effective strategies for addressing disparities in diabetes in rural and urban areas because of the differences in risk factors for type 1 and type 2 diabetes. Second, the sample size in some states might have been too small to detect significant ORs of diabetes in rural versus urban areas. This may lead to increased variability in estimates and reduced statistical power to determine meaningful differences. Third, because of the small sample sizes of the individual race categories included in the other race category (which included Asian, American Indian and Alaska Native, and multiracial) in most states, we were not able to separate these races into individual categories. Instead, we included the aggregated group of other races when adjusting the regressions models. Fourth, BRFSS uses telephone surveys, potentially leading to sampling bias. People, particularly those residing in rural areas who do not have telephones, have poor telecommunication service, or are less likely to answer telephone calls, may be underrepresented in the survey sample, affecting the generalizability of findings. Lastly, diabetes status was defined based on self-reported information, potentially underestimating the number of people living with diabetes. The rates of undiagnosed diabetes may be higher in rural areas (26,27).

### Conclusion

Our study examined the ORs of diabetes in rural versus urban areas at the state level and identified potential factors that contribute to the differences. Results of this analysis highlight the need for establishing effective policies to lower risk of diabetes and improve the quality of and access to diabetes prevention and care in rural areas. Understanding of the impact of nonmodifiable and modifiable risk factors on these differences might be crucial for developing more effective strategies to reduce health disparities between rural and urban communities.
